# Public awareness and attitudes toward palliative care in Northern Ireland

**DOI:** 10.1186/1472-684X-12-34

**Published:** 2013-09-17

**Authors:** Sonja McIlfatrick, Felicity Hasson, Dorry McLaughlin, Gail Johnston, Audrey Roulston, Lesley Rutherford, Helen Noble, Sheila Kelly, Avril Craig, W George Kernohan

**Affiliations:** 1 All Ireland Institute of Hospice and Palliative Care (AIIHPC) & Lead of the Palliative Care Forum for Northern Ireland, University of Ulster, Ulster, Northern Ireland; 2 Institute of Nursing and Health Research, University of Ulster, Ulster, Northern Ireland; 3 School of Nursing and Midwifery, Queen’s University Belfast, Belfast, UK; 4 School of Sociology, Social Policy, and Social Work, Queen’s University Belfast, Belfast, UK; 5 Marie Curie Hospice Belfast, Marie Curie Cancer Care, Belfast, UK; 6 Patient and Client Council Northern Ireland, Belfast, Northern Ireland; 7 Health and Social Care Research and Development Division, Public Health Agency, Northern Ireland, UK

**Keywords:** Palliative care, General public, Awareness, Questionnaire, Survey

## Abstract

**Background:**

The World Health Organisation recognises palliative care as a global public health issue and this is reflected at strategic level. Despite this, palliative care may not be universally welcomed. Surveys over the last decade have suggested that the general public have a lack of knowledge and negative perceptions towards palliative care. A detailed and comprehensive understanding of public views is needed in order to target education and policy campaigns and to manage future needs, expectations and resourcing of end of life care. The aim of this study was to establish the current levels of awareness and attitudes towards palliative care among the general public in Northern Ireland.

**Methods:**

A community-based cross-sectional survey with a population of 3,557 individuals aged over 17 years was performed. Information was collected using a structured questionnaire consisting of 17 items. Open questions were subject to content analysis; closed questions were subject to descriptive statistics with inferential testing as appropriate.

**Results:**

A total of 600 responses were obtained (response rate 17%). Responses indicated limited knowledge about palliative care. Female gender and previous experience influenced awareness in a positive direction. Respondents who worked in healthcare themselves or who had a close relative or friend who had used a palliative care service were more aware of palliative care and the availability of different palliative care services. Findings reveal the preferred place of care was the family home. The main barriers to raising awareness were fear, lack of interaction with health services and perception of lack of resources. A number of strategies to enhance awareness, access and community involvement in palliative care were suggested.

**Conclusions:**

Public awareness of the concept of palliative care and of service availability remains insufficient for widespread effective and appropriate palliative care to be accepted as the norm. In particular, those without previous family-related experiences lack awareness. This has implications for palliative care service provision and policy. An increased awareness of palliative care is needed, in order to improve knowledge of and access to services when required, empower individuals, involve communities and ultimately to realise the objectives contained within international strategies for palliative and end-of-life care.

## Background

The World Health Organisation [1] has advocated palliative care as being a public health issue: access to such care is acknowledged by United Nations conventions as a human right [2]. Although many governments generally adhere to this claim, placing palliative care within public health strategies, translation into practice varies widely. Awareness of palliative care varies widely according to international research to date [3-6] (Table 1). A telephone survey of 667 Irish adults [3] reported that many people were unfamiliar with the terms associated with palliative care and end of life. This low level of awareness may have implications for care, lead to negative impressions and impact on equitable access to services, resulting in negative consequences for the quality of care provided to the dying and bereaved [7-11]. European research suggests that this lack of awareness and recognition of palliative care among members of the general public is a key barrier to its future development [12]. Given that the western world’s population is aging, with greater incidence of cancer and a higher prevalence of chronic conditions [13,14], the demand for high quality hospice and palliative care services will undoubtedly increase. Within Europe, however, it has been noted that variations in access to care and care quality persist. Whilst the United Kingdom (UK) is acknowledged as leading in the development of palliative care services [15], historically there is a lack of understanding of the concept of palliative care and little is known about the awareness of the availability among members of the general public [16-19]. This is in contrast to the increasing profile of palliative care as a key national and regional strategic priority area [20-22]. Such initiatives have contributed to National Campaigns, such as Dying Matters, led by the National Council for Palliative Care [23] to specifically raise public awareness and change behaviour associated with death, dying and bereavement. To date, public awareness of palliative care, and what strategies should be used to target awareness, has received little attention from researchers. A detailed and comprehensive understanding of public views is needed in order to target education and policy campaigns and to manage future needs, expectations and resourcing of end of life care. Thus the aim of this study was to explore public views towards palliative care and explore strategies to improve awareness see.

**Table 1:** Summary of the key literature on public awareness of palliative care

**Author and year**	**Location**	**Sample**	**Method**	**Awareness level**
MacLeod et al. [24]	New Zealand	sampling matrix of 1011 adult subjects	Online survey	Findings revealed good understanding of the concept of palliative care, with 85% believing that palliative care staff provide comfort to people with terminal illness
Hirai et al. [4]	Japan	3984	Cross sectional anonymous questionnaire	Sixty-three per cent admitted no knowledge about palliative care
Benini et al. [25]	Italy	Random sample of 1897 adult subjects	Interviews	More than 40% had never heard of palliative care with only 23% declared having an adequate or precise idea of what PC is.
Australian Government Department of Health & Ageing [26]	Australia	Stratified sample of 1201 adult subjects	Mixed methods which included a telephone survey	Australians had a low to moderate knowledge and understanding of palliative care - 38% could explain palliative care to another, 33% only know a little, 13% have heard the term and 16% were not aware.
Claxton-Oldfield et al. [6]	Canada	Random sample 89 adult subjects	Face to face survey	Seventy-five per cent had heard of palliative care, however, only about half of these (48%) defined it as care for terminally ill or dying persons.
Wallace [16]	Scotland	Random sample 668 adult subjects	Telephone survey	Most reported some knowledge of palliative care (49%), with under a third reporting no knowledge.

## Methods

### Design

A cross-sectional design using a self-administered anonymous questionnaire survey (postal and online) was used to assess public awareness of palliative care. Data were collected over a four week period during November 2011. The three-part questionnaire was adapted from similar studies conducted in a number of other countries [16,26,27] to enable comparison (see questionnaire). With minor modifications the questionnaire consisted of both open and closed questions. Open-ended questions were included to gather subjective opinions about palliative care, barriers and strategies to promote uptake. The questionnaire was divided into three: part one elicited information on the respondent’s knowledge and attitude statements towards palliative care. Part two adopted an open question style to record up to three key barriers to palliative care promotion and strategies they would employ to overcome them. The final section recorded seven demographic characteristics. Face and content validation of the questionnaire was undertaken by a research advisory group consisting of experts in the area of palliative care.

### Sample

The target population was members of the Patient and Client Council for Northern Ireland. This organisation has been established to provide an independent voice for patients, clients, carers and communities on health and social care. The membership list acted as the sampling frame comprising names, dates of birth and contact details of 4,322 people who have expressed an interest to take part in research. For ethical reasons only members aged over 18 were included. The potential population consisted of 3,557 members. Sample size requirements were reviewed using a sample size calculator [28] and suggested that a sample of 347 was required to provide a confidence level of 95% in the primary outcome, self-assessed knowledge of palliative care.

An information pack (disseminated both online and by post) which contained a cover letter/participant information sheet, questionnaire and sources of support for further information was sent to 3,557 members. An online questionnaire was set up (http://www.surveymonkey.com) and remained open for four weeks. One reminder letter (postal and email) was sent one week after the questionnaire was disseminated and a reminder advert placed on the membership newsletter two weeks later. Respondents were informed that completion and return of questionnaires was taken to imply consent.

### Analysis

Descriptive statistics were obtained on responses to each question (IBM SPSS vs. 20.0). Chi-square tests were used where appropriate to determine the association between socio-demographic variables (which included gender, age) and the outcomes of interest (knowledge of palliative care). The association was considered statistically significant when p-values were less than 0.05. Open-ended questions from the questionnaire were content analysed for themes using Miles and Huberman’s [29] approach. This involved three stages, data reduction, display and conclusion drawing and verification. Two authors (FH and WK) analysed open questions.

### Ethical considerations

The benefits in gaining data to inform public education were deemed to outweigh the possible risks of possible inconvenience and potential distress, which was minimised through the use of sensitive questioning. Ethical approval for the study was obtained under arrangements for research governance at the University of Ulster. The survey approach assured confidentiality.

## Results

A total of 600 people responded, giving a 17% response rate. Whilst this is lower than anticipated, it does represent a substantial response and the sample size achieved was in excess of that required to provide a confidence level of 95% in the primary outcome, self-assessed knowledge of palliative care. The majority of participants were female (n = 413, 69%) and the modal age group was 60–69 years. Ethnic origin was mainly white (n = 549, 92%) and the most common religious denomination was Protestant (n = 249, 42%) followed by Roman Catholic (n = 194, 32%). Analysis revels that the majority of respondents were married and most declared their denomination as Protestant. The religion and ethnicity of participants broadly represent Northern Ireland’s population (see Table 2).

**Table 2:** Showing the range of demographic characteristics of respondents to the survey

	**Category**	**No (%)**
**Gender (%)**	Male	151 (25.2%)
	Female	413 (68.8%)
	Non response	36 (6.0%)
**Age (%)**	Under 20	3 (0.5%)
	20-39	58 (9.6%)
	40-59	214 (35.7%)
	60-70	268 (44.6%)
	80 and over	23 (3.8%)
	Non response	34 (5.7%
**Employed**	Employed	202 (33.7%)
	Non-employed	109(18.3%)
	Retired	252 (42%)
	Non response	37 (6.2%)
**Marital status**	Single, never married	85 (14.2%)
	Cohabiting	9 (1.5%)
	Married	324 (54%)
	Separated	21 (3.5%)
	Divorced	38 (6.3%)
	Widow/Widower	78 (13%)
	Non Response	45 (7.5%)
**Religion**	Protestant	249 (41.5%)
	Catholic	194 (32.3%)
	Other Christian denomination	23 (3.8%)
	Jewish, Hindu Buddhist Muslim	4 (0.7%)
	Humanist	11 (1.8%)
	Atheist	15 (2.5%)
	Agnostic	13 (2.2%)
	Other religion or belief system	10 (1.7%)
	Prefer not to say	36 (6%)
	Non response	45 (7.5%)
**Ethnic Origin**	White	549 (91.5%)
	Mixed	4 (0.7%)
	Indian; Black African	4 (0.7%)
	No response	43(7.2%)

### Knowledge of palliative care

Whilst the majority of respondents reported that they had heard the term palliative care (n = 500, 83%), most people revealed they had little or no knowledge of its meaning: over half (n = 336, 56%) claimed to have low knowledge whilst a fifth (n = 114, 19%) stated they had no understanding of the concept of palliative care (see Table 3). Women reported higher levels of knowledge than men. There was a statistically significant difference between males and females in knowledge of palliative care (p = 0.01 Mann–Whitney, 2 tailed) and older age groups reported higher levels of knowledge than younger people (Nonpar. Correlation, Spearman’s rho, p = 0.005).

**Table 3:** Showing limited level of self-assessed knowledge of “palliative care” in the general population

	***No %***
No knowledge	114 (19%)
Some knowledge	336 (56%)
Quite a bit of knowledge	96 (16%)
Very knowledgeable	26 (4.3%)
Missing/ not applicable	28 (4.7%)

When asked to define what palliative care meant, responses tended to be fairly broad in nature, with only some participants demonstrating a detailed knowledge of specific aspects of palliative care: notably those who had worked in a heath care setting. The majority defined palliative care as pain relief for people with terminal illness at the end of life with the aim of achieving a peaceful death. Although participants were not asked to specify conditions, many associated palliative care with cancer and care of older people.

*“It is making a person who has terminal cancer as comfortable and as pain-free as possible as end of life approaches”* (Res. 16)

*“Palliative care is a health unit which provides care and treatment for cancer patients”* (Res. 112)

When respondents were asked to reflect on the aims of palliative care, the majority cited delivery of comfort (n = 492, 82%), pain relief (n = 488, 81.3%) and dignity (n = 458, 76.3%) as being the key aims.

In terms of preferred place of the care, the family home (n = 366, 61%), was most often identified as the preferred place of care for a patient with palliative care needs, followed by the hospice (n = 41 6.8%), hospital (n = 14, 2.3%) and nursing home (n = 5, 0.8%). Of note, however, was that 17.5% (n = 105) identified a combination of hospital, hospice and home as their preferred place of care. When asked where the participants believed palliative care was delivered the majority believed it was delivered in hospice and at home (n = 461, 76.8%) followed by hospital (n = 349, 58.2%). Furthermore, the majority of respondents believed that a member of the generalist practice team (general practitioner or district nurse) or a specialist hospice nurse (n = 367, 61.2%) would be best placed to discuss palliative care needs.

### Source of information

The top three key sources of information on palliative care that were declared were (see Table 4):

(1) through a close friend or relative who had received care;

(2) via their work in a health care setting;

(3) through newspapers and magazines.

**Table 4:** Range of sources of information about “palliative care” among 600 members of the general public of Northern Ireland surveyed in 2011

**Category**	***N (%)***
Close friend / relative received Palliative Care	262 (43.7%)
You work in a health care setting	143 (23.8%)
Newspaper/Magazine	123 (20.5%
Television	117 (19.5%)
Distant friend / relative received Palliative Care	90 (15%)
Radio	84 (14%)
Friend discussed it	72 (12%)
Relative discussed it	66 (11%)
People collecting money	63(10.5%)
Neighbour received Palliative Care	53 (8.8%)
Internet/Social Media	35 (5.8%)
Not sure/ can’t remember	29 (4.8%)
You work in Palliative Care	21 (3.5%)
Have personally received Palliative Care	14(2.3%)

In addition, a small number of respondents had themselves received palliative care. Having such direct (n = 14) or vicarious (n = 405) experience in the past was a very strong indicator of level of knowledge (χ^2^, p < 0.001). Similarly, working in a health care setting was strongly associated with knowledge of palliative care (χ^2^, p < 0.001).

When asked which source they considered the most important, respondents identified the media (television, radio, newspapers) as key in delivering messages to the general public. Interestingly, however this was only recorded as the third source actually used.

### Barriers to improving awareness

Respondents were asked to record three key barriers they felt hindered awareness of palliative care among the general public. Despite recognising the importance of having the opportunity to talk, the overall barrier cited was the reluctance among the general public to talk about death and dying. This was attributed to fear and a taboo within society to openly address such issues, which was aligned with not knowing what to do and a fear of causing upset to friends and family.

The second barrier was the lack of knowledge or information held about palliative care. Many reported they were unaware of the reality of death, the processes of dying and grieving which led to the general public making assumptions which were reinforced by a lack of continuity and coordination between health care services. One common example of this was the lack of discussion of palliative care at diagnosis or when treatment starts. Respondents noted that palliative care was introduced at the end of the patients’ journey. Palliative care was equated with hospice and end of life care, caring mainly for older patients or those suffering from cancers. The third barrier was attributed to a lack of funding and resources dedicated to promoting palliative care and engaging with communities. Perceptions that hospice played a key role in the delivery of palliative care led to the general public assuming that treatment choices were limited to urban populations that are proximate to a hospice.

### Strategies to improve awareness and access

Respondents were asked to record strategies they felt could enhance awareness, access and community involvement in palliative care. Findings revealed a range of approaches, such as publicity campaigns, posters, talks, open days and clear signposting from health professionals, suggested to enhance awareness. Access to palliative care services was perceived to be dependent upon the structure of generic and specialist health care services, with access to palliative care services requiring greater collaboration with generic and specialist services working as one in the referral process. It was also suggested that access could be improved by health and social care professionals being made more aware of the availability of services and having a role in communicating the availability of such services to patients. Finally, the issue of the location of palliative care services, mainly perceived as being urban-based, highlighted the potential for greater transportation services to be available to address the needs of more rural dwellers.

## Discussion

The results of this study are in line with the emerging international literature on public awareness of palliative care, which has suggested variable (or inadequate) awareness of palliative care among the general public [5,6,16]. This variability extends across studies undertaken over the last decade in countries such as Canada, United Kingdom, Europe, Japan, Australia and New Zealand. It could be argued, that there does appear to be some change in recognition of the importance of public awareness of palliative care in adopting a public health approach to palliative care. According to Paul and Snallow [30] this has been driven by policy, practice and theoretical literature both within the UK and globally. The theoretical basis for the public health approach to palliative care has been led and developed in Australia by the work of Kellehear and O’Connor [31] and Kellehear [32]. Various international examples from India [33], Australia [31]; and the UK [34], have clearly demonstrated the importance of such approaches in increasing access to palliative care services and improving understanding and awareness. This remains however a largely under-researched area, despite being recognised as a significant policy priority. Further research is needed to examine the impact and evidence for such interventions, hence providing further clarity on the public health approach to palliative care.

The findings in this study indicated that while many members of the public had heard of the concept of palliative care, there was a clear lack of familiarity and awareness of what palliative care really means. A repeated theme, borne out in this study, was the key influence of having a close friend or relative accessing palliative care services upon increased awareness and familiarity with the concept and services. There was a general tendency to identify palliative care goals in line with pain relief to provide comfort and dignity at the end of life to elderly cancer patients. Respondents emphasised the patient’s clinical needs. Yet this is in stark contrast to the World Health Organisation [1] advocating that palliative care should not only improve the quality of life for patients but also for their families, being available from diagnosis regardless of condition or age. This is important to consider when set against the backdrop that over the last decade in the UK, there has been considerable government and media attention devoted to raising public awareness of palliative care.

Of significance is the discussion around preferred place of care. The findings from this study indicate that the vast majority of respondents were aware that palliative care would be delivered across a range of health care settings such as hospice, and hospital, however the preferred place of care for a patient with palliative care needs was identified as the family home. Achievement of preferred place of care for terminally ill individuals has increasingly been used as a quantifiable indicator of the effectiveness of palliative care services [35]. Whilst population studies have indicated that the majority of people would prefer to die at home, secular trends towards an institutionalised death have been reported in many countries [36]. In expanding this debate it is important to note that whilst home represents the preferred place of care and death for the majority of terminally ill individuals [37], this wish was certainly not shared by all. These factors contribute to a much wider debate on the provision of palliative care as an international human right, promoting choice, autonomy and equity of access to services for all, without discrimination [38].

The perceived barriers to raising awareness of palliative care centred upon social and cultural taboos and limited resources. Perhaps of greatest interest is the recognition among the majority in the sample of a lack of coordinated approach and communication among health and social care service providers as a key barrier. This insight may be a contributing factor to variability of awareness of palliative care, impacting on perceptions and use by those patients and families who require it. This indicates that raising public awareness cannot be undertaken in isolation but requires the concept of palliative care to be brought forward within the delivery of health care systems.

### Limitations

Whilst this study represents one of the largest recent studies exploring awareness of palliative care among the general public it has a number of limitations: bias relating to the low response rate (17%) and accessing a population that was self-selected by its explicit interest in cooperation in health and social care research. The true picture of public awareness may be even lower than data suggests. Secondly, while the questionnaire was based on a review of the literature and subject to content validity by experts it was not tested formally for reliability. This is particularly relevant to estimates of attitude. Finally, whilst the use of open questions within the questionnaire allowed respondents to express themselves in their own words, different respondents provided different degrees of detail, questioning the comprehensiveness of the results.

## Conclusions

Whilst the majority of respondents had heard of the term palliative care, findings revealed inadequate understanding of the concept. Low levels of understanding highlight the need for a parallel approach ensuring that palliative care is more integrated into health service systems alongside a continued public health approach to palliative care in order to eradicate social taboos and ensure such services are sought out when required. Future research should focus on mechanisms to integrate palliative care more fully into the health system, contribute to the conceptual basis for public health approaches to palliative care and the evaluation of different strategies to increase public awareness and understanding of palliative care.

## Competing interests

No competing financial or non-financial interests exist.

## Authors’ contributions

All authors were involved in the study conception and design. SMcI, FH, AC, WG, SK were involved in data collection, FH & WK were involved in data analysis. All authors were involved in drafting of manuscript and critical revision of the manuscript. All authors read and approved the final manuscript.

## Pre-publication history

The pre-publication history for this paper can be accessed here:

http://www.biomedcentral.com/1472-684X/12/34/prepub
